# Daratumumab, lenalidomide, and dexamethasone in relapsed/refractory myeloma: a cytogenetic subgroup analysis of POLLUX

**DOI:** 10.1038/s41408-020-00375-2

**Published:** 2020-11-03

**Authors:** Jonathan L. Kaufman, Meletios A. Dimopoulos, Darrell White, Lotfi Benboubker, Gordon Cook, Merav Leiba, James Morton, P. Joy Ho, Kihyun Kim, Naoki Takezako, Philippe Moreau, Heather J. Sutherland, Hila Magen, Shinsuke Iida, Jin Seok Kim, H. Miles Prince, Tara Cochrane, Albert Oriol, Nizar J. Bahlis, Ajai Chari, Lisa O’Rourke, Sonali Trivedi, Tineke Casneuf, Maria Krevvata, Jon Ukropec, Rachel Kobos, Hervé Avet-Loiseau, Saad Z. Usmani, Jesus San-Miguel

**Affiliations:** 1 Winship Cancer Institute, Emory University, Atlanta, GA USA; 2 The National and Kapodistrian University of Athens, Athens, Greece; 3 Dalhousie University and Queen Elizabeth II Health Sciences Centre, Halifax, Nova Scotia Canada; 4 Service d’Hématologie et Thérapie Cellulaire, Hôpital Bretonneau, Centre Hospitalier Régional Universitaire, Tours, France; 5 St James’s Institute of Oncology, Leeds Teaching Hospitals National Health Service Trust and University of Leeds, Leeds, UK; 6 Assuta Ashdod University Hospital, Faculty of Health Science Ben-Gurion University of the Negev, Beer Sheva, Israel; 7 Icon Cancer Care, South Brisbane, QLD Australia; 8 Institute of Haematology, Royal Prince Alfred Hospital, Camperdown, NSW Australia; 9 Department of Medicine, Samsung Medical Center, Sungkyunkwan University School of Medicine, Seoul, South Korea; 10 Department of Hematology, National Hospital Organization Disaster Medical Center of Japan, Tachikawa, Japan; 11 Hematology, University Hospital Hôtel-Dieu, Nantes, France; 12 Leukemia/Bone Marrow Transplant Program, University of British Columbia, Vancouver, Canada; 13 Department of Hematology Chaim Sheba Medical Center, Ramat-Gan, Sackler Faculty of Medicine, Tel Aviv University, Tel Aviv, Israel; 14 Department of Hematology and Oncology, Nagoya City University Graduate School of Medical Sciences, Nagoya, Japan; 15 Yonsei University College of Medicine, Severance Hospital, Seoul, South Korea; 16 Cabrini Hospital, Epworth HealthCare and Sir Peter MacCallum Department of Oncology, University of Melbourne, Melbourne, VIC Australia; 17 Gold Coast University Hospital and Griffiths University, Southport, QLD Australia; 18 Institut Català d’Oncologia i Institut Josep Carreras, Hospital Germans Trias I Pujol, Barcelona, Spain; 19 University of Calgary, Arnie Charbonneau Cancer Institute, Calgary, AB Canada; 20 Icahn School of Medicine at Mount Sinai, New York, NY USA; 21 Janssen Research & Development, Spring House, PA USA; 22 Janssen Research & Development, Beerse, Belgium; 23 Janssen Global Medical Affairs, Horsham, PA USA; 24 Janssen Research & Development, Raritan, NJ USA; 25 Unite de Genomique du Myelome, IUC-Oncopole, Toulouse, France; 26 Levine Cancer Institute/Atrium Health, Charlotte, NC USA; 27 Clínica Universidad de Navarra-Centro de Investigación Médica Aplicada, Instituto de Investigación Sanitaria de Navarra, Centro de Investigación Biomédica en Red de Cáncer, Pamplona, Spain

**Keywords:** Medical research, Cancer

## Abstract

High cytogenetic risk abnormalities confer poor outcomes in multiple myeloma patients. In POLLUX, daratumumab/lenalidomide/dexamethasone (D-Rd) demonstrated significant clinical benefit versus lenalidomide/dexamethasone (Rd) in relapsed/refractory multiple myeloma (RRMM) patients. We report an updated subgroup analysis of POLLUX based on cytogenetic risk. The cytogenetic risk was determined using fluorescence in situ hybridization/karyotyping; patients with high cytogenetic risk had *t*(4;14), *t*(14;16), or del17p abnormalities. Minimal residual disease (MRD; 10^–5^) was assessed via the clonoSEQ^®^ assay V2.0. 569 patients were randomized (D-Rd, *n* = 286; Rd, *n* = 283); 35 (12%) patients per group had high cytogenetic risk. After a median follow-up of 44.3 months, D-Rd prolonged progression-free survival (PFS) versus Rd in standard cytogenetic risk (median: not estimable vs 18.6 months; hazard ratio [HR], 0.43; *P* < 0.0001) and high cytogenetic risk (median: 26.8 vs 8.3 months; HR, 0.34; *P* = 0.0035) patients. Responses with D-Rd were deep, including higher MRD negativity and sustained MRD-negativity rates versus Rd, regardless of cytogenetic risk. PFS on subsequent line of therapy was improved with D-Rd versus Rd in both cytogenetic risk subgroups. The safety profile of D-Rd by cytogenetic risk was consistent with the overall population. These findings demonstrate the improved efficacy of daratumumab plus standard of care versus standard of care in RRMM, regardless of cytogenetic risk.

## Introduction

Daratumumab is a human IgGκ monoclonal antibody targeting CD38 with a direct on-tumor^1–4^ and immunomodulatory^5–7^ mechanism of action. Intravenous daratumumab 16 mg/kg is approved in the USA in combination with lenalidomide and dexamethasone (Rd) or bortezomib and dexamethasone (Vd) in patients with multiple myeloma (MM) who received at least one prior line of therapy, in combination with bortezomib/melphalan/prednisone or Rd in patients with transplant-ineligible newly diagnosed MM, and in combination with bortezomib, thalidomide, and dexamethasone in patients with transplant-eligible newly diagnosed MM^8^. Daratumumab is also approved in the USA as monotherapy in patients with heavily pretreated relapsed or refractory MM (RRMM) and in combination with pomalidomide/dexamethasone for patients with at least two prior therapies, including lenalidomide and a proteasome inhibitor^8^.

In the phase 3 POLLUX study in patients with RRMM, adding daratumumab to Rd (D-Rd) more than doubled complete response (CR) or better rates, induced a > four-fold increase in the rate of minimal residual disease (MRD) negativity at the 10^–5^ sensitivity threshold, and reduced the risk of disease progression or death by 63% versus Rd alone at a median follow-up of 13.5 months^9^. With longer follow-up (median: 44.3 months), D-Rd continued to reduce the risk of disease progression or death by 56% and significantly increased the overall response rate (ORR; 93% vs 76%; *P* < 0.0001) and rates of CR or better (57% vs 23%; *P* < 0.0001), very good partial response (VGPR) or better (80% vs 49%; *P* < 0.0001), and MRD negativity (10^–5^ sensitivity threshold; 30% vs 5%; *P* < 0.000001) versus Rd alone^10^.

Patients with MM and high cytogenetic risk abnormalities including IgH translocations (e.g., *t*[4;14]) and genomic imbalance (e.g., del17p) have worse progression-free survival (PFS) and overall survival outcomes compared with patients with standard cytogenetic risk^11,12^. Although high cytogenetic risk abnormalities are most relevant as a prognostic marker prior to relapse, several studies have shown that they have a negative effect on survival for RRMM^11,12^. The 2009 International Myeloma Working Group (IMWG) guidelines define high cytogenetic risk as having at least one of the following abnormalities: *t*(4;14), *t*(14;16), or del17p, determined by fluorescence in situ hybridization (FISH)^13^. More recently, and after the POLLUX study was initiated, updated IMWG guidelines were published in 2016, wherein the definition of high cytogenetic risk was expanded to include *t*(14;20) and gain1q abnormalities^11^. In patients with high cytogenetic risk, treatment choice is driven by the duration, quality, and depth of response offered; updated IMWG guidelines recommend treatment with regimens containing bortezomib or lenalidomide in these patients.

After a median follow-up of 25.4 months, D-Rd significantly prolonged PFS and increased ORR in patients with RRMM in POLLUX; improvement was also seen regardless of cytogenetic risk status^14^. Here, we report the updated efficacy and safety findings after a median follow-up of more than 3 years for patients with standard and high cytogenetic risk RRMM.

## Methods

### Patients

A total of 439 patients who underwent cytogenetic testing from phase 3 POLLUX study (ClinicalTrials.gov identifier: NCT02076009) were included in this analysis. The study design, complete eligibility criteria, and primary and subgroup analysis results have been previously published^9,14^. Briefly, eligible patients were ≥18 years of age with an Eastern Cooperative Oncology Group performance status score of 0 to 2. Patients had received at least one prior line of therapy for MM, achieved at least a partial response to at least one prior MM therapy, and had documented evidence of progressive disease based on IMWG criteria on or after their last regimen. Patients were excluded if they were refractory to or intolerant of lenalidomide or had a creatinine clearance of <30 mL/min/1.73 m^2^.

### Study design and treatment

POLLUX is a phase 3, randomized, open-label, active-controlled, multicenter study in patients with RRMM. Randomization was stratified according to International Staging System (ISS) disease stage (I vs II vs III) at screening, number of prior lines of therapy (1 vs 2 or 3 vs >3), and prior lenalidomide exposure (no vs yes). Exploratory analyses were conducted for subgroups of patients based on cytogenetic risk status. Patients were randomized 1:1 to Rd (lenalidomide: 25 mg orally on Days 1–21 of each 28-day cycle; dexamethasone: 40 mg orally weekly) with or without daratumumab (16 mg/kg IV weekly for 8 weeks, every 2 weeks for 16 weeks, and then every 4 weeks) until progression.

### Cytogenetic risk evaluation

Cytogenetic abnormalities were detected by local FISH or karyotyping on bone marrow aspirates collected at screening visits. Determination of each abnormality and threshold of frequencies to consider a positive finding was determined locally and varied by site. Patients in the intent-to-treat (ITT) population who had at least one assessment from FISH or karyotyping were included in the analysis. Patients with high cytogenetic risk status had at least one of the following cytogenetic abnormalities identified: *t*(4;14), *t*(14;16), or del17p.

### MRD evaluation

MRD was assessed at the time of the suspected CR (including stringent CR; blinded to treatment group), and if CR was maintained, at 3 months, 6 months, and every 12 months after confirmation of CR. MRD testing was performed via the clonoSEQ^®^ assay V2.0 (Adaptive Biotechnologies, Seattle, WA, USA) at the 10^–5^ sensitivity threshold (one cancer cell per 100,000 nucleated cells). Patients with an MRD-negative test result were considered to be MRD negative, and patients with only MRD-positive or indeterminate test results or who had not undergone MRD testing were considered to be MRD positive. Sustained MRD negativity was defined as confirmed maintenance of MRD-negative status at a sensitivity threshold of 10^–5^ for ≥6 or ≥12 months.

### Statistical analyses and assessments

PFS analyses for the cytogenetic risk groups included patients in the ITT population who met the biomarker criteria for risk assessment. Patients with measurable disease at baseline or at the screening visit who received at least one study treatment and had undergone at least one post-baseline disease assessment were included in the response-evaluable population. Patients who received at least one administration of study treatment were included in the safety population.

PFS and time to response were compared between the D-Rd and Rd treatment groups using a stratified log-rank test. Hazard ratios (HRs) and 95% confidence intervals (CIs) were estimated using a Cox proportional hazards model with treatment as the sole explanatory variable. The Kaplan–Meier method was used to estimate the distributions. PFS on the subsequent line of therapy (PFS2) was defined as the time from randomization to disease progression after the next line of subsequent therapy or death. Differences in ORRs, rates of CR or better, and rates of VGPR or better between treatment groups were measured using a stratified Cochran–Mantel–Haenszel chi-square test.

MRD and sustained MRD negativity were evaluated for patients in the entire ITT population who met the biomarker criteria for risk assessment to allow for a stringent and unbiased evaluation of MRD negativity. MRD-negativity rates were defined as the proportion of patients who achieved MRD-negative status at any time point following the first treatment dose. A Fisher’s exact test was used to compare MRD-negativity rates between the D-Rd and Rd treatment groups.

### Study oversight

Institutional review boards or ethics committees approved the research at each clinical study site. All patients provided written informed consent. The study design and analyses were devised by the investigators and sponsor, and study data were collected by the investigators and their research teams. Final data analysis and verification of accuracy were conducted by Janssen. The investigators were not restricted by confidentiality agreements and had full accessibility to all the data. Writing assistance was funded by Janssen Global Services. The study was sponsored by Janssen Research & Development, LLC and was registered at ClinicalTrials.gov (NCT02076009).

### Data-sharing statement

The data-sharing policy of Janssen Pharmaceutical Companies of Johnson & Johnson is available at https://www.janssen.com/clinical-trials/transparency. As noted on this site, requests for access to the study data can be submitted through the Yale Open Data Access Project site at http://yoda.yale.edu.

## Results

### Patients and treatments

A total of 569 patients were randomized, with 286 assigned to D-Rd and 283 to Rd. A total of 439 (77%) patients underwent cytogenetic testing; 324 (57%) patients were evaluated using FISH, 261 (46%) patients were evaluated using karyotyping, and 146 (26%) were evaluated using both. Of these, high cytogenetic risk abnormalities were reported in 35 (12%) patients in the D-Rd group and 35 (12%) patients in the Rd group. The standard cytogenetic risk was reported in 193 (68%) patients in the D-Rd group and 176 (62%) patients in the Rd group. Among patients assessed for cytogenetic risk, patient demographics, baseline disease, and clinical characteristics based on cytogenetic status are shown in Table 1. Among patients achieving CR or better, MRD was not evaluated in 48 (22%) patients. At the time of the clinical cutoff (October 10, 2018), 253 (69%) and 57 (83%) patients discontinued treatment in the standard and high cytogenetic risk subgroups, respectively (Table 2).

**Table 1:** Patient demographics, baseline disease, and clinical characteristics.

	Standard cytogenetic risk*		High cytogenetic risk*^,†^	
Characteristic	D-Rd (*n* = 193)	Rd (*n* = 176)	D-Rd (*n* = 35)	Rd (*n* = 35)
Age, y				
Median (range)	66 (36–89)	64 (42–85)	67 (50–80)	67 (50–81)
≥75 y, *n* (%)	21 (11)	17 (10)	4 (11)	6 (17)
Sex, *n* (%)				
Male	114 (59)	102 (58)	19 (54)	19 (54)
Race, *n* (%)				
White	131 (68)	120 (68)	28 (80)	23 (66)
Asian	47 (24)	35 (20)	4 (11)	8 (23)
Black or African American	4 (2)	5 (3)	1 (3)	2 (6)
Unknown/not reported	11 (6)	16 (9)	2 (6)	2 (6)
ISS stage,^‡^ *n* (%)				
I	96 (50)	92 (52)	13 (37)	14 (40)
II	62 (32)	50 (28)	15 (43)	13 (37)
III	35 (18)	34 (19)	7 (20)	8 (23)
ECOG performance status score, *n* (%)				
0	94 (49)	90 (51)	15 (43)	22 (63)
1	91 (47)	78 (44)	18 (51)	12 (34)
2	8 (4)	8 (5)	2 (6)	1 (3)
Cytogenetic profile,*^,†^ *n* (%)				
*t*(4;14)	–	–	10 (29)	15 (43)
*t*(14;16)	–	–	2 (6)	6 (17)
del17p	–	–	25 (71)	20 (57)
≥2 risk factors^§^	–	–	2 (6)	5 (14)
Median (range) time from diagnosis, y	3.7 (0.6–22.5)	4.0 (0.4–18.3)	3.2 (0.4–27.0)	2.3 (0.4–14.6)
Prior lines of therapy, *n* (%)				
1	96 (50)	90 (51)	22 (63)	20 (57)
2	62 (32)	47 (27)	6 (17)	9 (26)
3	24 (12)	24 (14)	5 (14)	4 (11)
>3	11 (6)	15 (9)	2 (6)	2 (6)
Median (range)	2 (1–11)	1 (1–8)	1 (1–6)	1 (1–6)
Prior ASCT, *n* (%)	115 (60)	115 (65)	22 (63)	19 (54)
Prior PI, *n* (%)	162 (84)	148 (84)	31 (89)	31 (89)
Bortezomib	160 (83)	145 (82)	30 (86)	31 (89)
Prior IMiD, *n* (%)	103 (53)	102 (58)	22 (63)	15 (43)
Lenalidomide	27 (14)	33 (19)	10 (29)	4 (11)
Prior PI + IMiD, *n* (%)	79 (41)	80 (46)	18 (51)	12 (34)
Refractory to PI only, *n* (%)	42 (22)	29 (17)	8 (23)	9 (26)
Refractory to IMiD only, *n* (%)	7 (4)	10 (6)	3 (9)	1 (3)
Refractory to PI and IMiD, *n* (%)	6 (3)	7 (4)	1 (3)	3 (9)
Refractory to last line of therapy, *n* (%)	62 (32)	50 (28)	11 (31)	13 (37)

*D-Rd* daratumumab plus lenalidomide/dexamethasone, *Rd* lenalidomide/dexamethasone, *ISS* International Staging System, *ECOG* Eastern Cooperative Oncology Group, *ASCT* autologous stem cell transplant, *PI* proteasome inhibitor, *IMiD* immunomodulatory drug, *FISH* fluorescence in situ hybridization.

Note: percentages may not equal 100% due to rounding.

*Based on FISH/karyotyping.

^†^Patients with high cytogenetic risk had a *t*(4;14), *t*(14;16), or del17p abnormality.

^‡^ISS stage is derived based on the combination of serum β2-microglobulin and albumin.

^§^Patients with ≥2 of the *t*(4;14), *t*(14;16), or del17p risk factors.

**Table 2:** Patient disposition based on cytogenetic risk* status.

	Standard risk		High risk^†^	
Treatment discontinuation,^‡^ *n* (%)	D-Rd (*n* = 192)	Rd (*n* = 176)	D-Rd (*n* = 35)	Rd (*n* = 34)
Patients who discontinued treatment	105 (55)	148 (84)	26 (74)	31 (91)
Reason for discontinuation				
Progressive disease	57 (30)	104 (59)	17 (49)	25 (74)
Adverse event	28 (15)	22 (13)	7 (20)	4 (12)
Noncompliance with study drug^§^	7 (4)	4 (2)	0	2 (6)
Withdrawal by patient	2 (1)	8 (5)	1 (3)	0
Physician decision	6 (3)	3 (2)	1 (3)	0
Death	3 (2)	5 (3)	0	0
Other	2 (1)	2 (1)	0	0

*D-Rd* daratumumab plus lenalidomide/dexamethasone, *Rd* lenalidomide/dexamethasone, *FISH* fluorescence in situ hybridization.

*Based on FISH/karyotyping.

^†^Patients with high cytogenetic risk had a *t*(4;14), *t*(14;16), or del17p abnormality.

^‡^Safety population.

^§^Based on reason, “patient refused to further study treatment” at “end of treatment.”

### Updated efficacy results

**Fig. 1:**
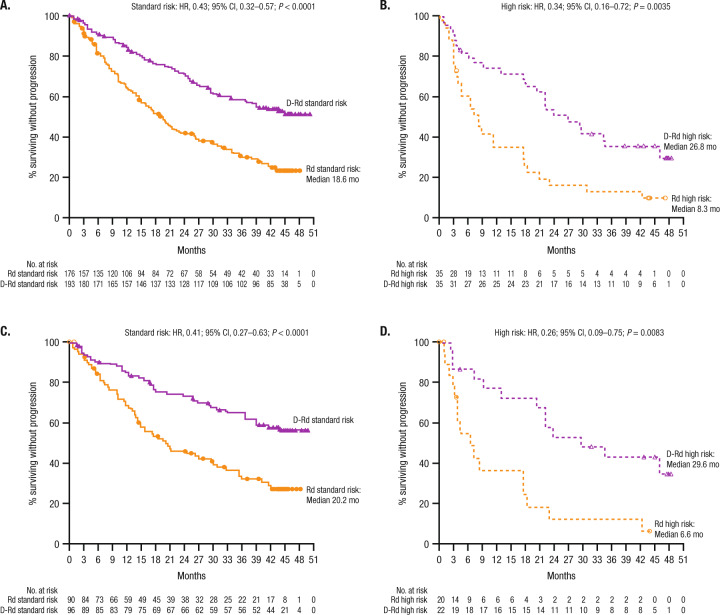
PFS based on cytogenetic risk status. PFS in the ITT/biomarker risk population:* **A** standard and **B** high cytogenetic risk patients. PFS in patients with one prior line of therapy: **C** standard and **D** high cytogenetic risk patients. PFS progression-free survival, HR hazard ratio, CI confidence interval, D-Rd daratumumab plus lenalidomide/dexamethasone, Rd lenalidomide/dexamethasone, ITT intent-to-treat. *Patients in the ITT population who met the biomarker criteria for risk assessment.

After a median follow-up of 44.3 months, D-Rd prolonged PFS versus Rd in patients with standard cytogenetic risk (median: not estimable [NE] vs 18.6 months; HR, 0.43; 95% CI, 0.32–0.57; *P* < 0.0001; Fig. 1A) and high cytogenetic risk (median: 26.8 months vs 8.3 months; HR, 0.34; 95% CI, 0.16–0.72; *P* = 0.0035; Fig. 1B) in the ITT population. The 42-month PFS rates were 53% with D-Rd versus 25% with Rd in the standard cytogenetic risk subgroup and 35% versus 13%, respectively, in the high cytogenetic risk subgroup. Among patients with 1 prior line of therapy, D-Rd prolonged PFS versus Rd in patients with standard cytogenetic risk (median: NE vs 20.2 months; HR, 0.41; 95% CI, 0.27–0.63; *P* < 0.0001; 42-month PFS rate: 58% vs 27%; Fig. 1C) and high cytogenetic risk (median: 29.6 months vs 6.6 months; HR, 0.26; 95% CI, 0.09–0.75; *P* = 0.0083; 42-month PFS rate: 43% vs 12%; Fig. 1D).

The ORR and rates of VGPR or better and CR or better were higher with D-Rd compared with Rd, regardless of cytogenetic risk status (Table 3). Among patients with standard cytogenetic risk, time to VGPR or better (median: 3.7 vs 8.3 months; HR, 1.98; 95% CI, 1.52–2.59; *P* < 0.0001) and CR or better (median: 13.9 months vs NE; HR, 2.38; 95% CI, 1.66–3.43; *P* < 0.0001) were decreased with D-Rd versus Rd. Time to VGPR or better (median: 3.7 months vs 18.7 months; HR, 3.45; 95% CI, 1.41–8.39; *P* = 0.0040) and CR or better (median: 14.4 months vs NE; HR, 2.07; 95% CI, 0.51–8.48; *P* = 0.3011) were also decreased with D-Rd versus Rd for patients with high cytogenetic risk.

**Table 3:** Response and MRD-negativity rates in patients with standard and high cytogenetic risk.

	Standard risk			High risk*		
Response,^†^ *n* (%)	D-Rd (*n* = 190)	Rd (*n* = 172)	*P*	D-Rd (*n* = 35)	Rd (*n* = 34)	*P*
ORR	178 (94)	135 (79)	<0.0001	31 (89)	23 (68)	0.0145
≥CR	111 (58)	43 (25)		15 (43)	3 (9)	
Stringent CR	61 (32)	23 (13)		10 (29)	1 (3)	
CR	50 (26)	20 (12)		5 (14)	2 (6)	
≥VGPR	156 (82)	92 (54)	<0.0001	25 (71)	10 (29)	0.0004
VGPR	45 (24)	49 (29)		10 (29)	7 (21)	
PR	22 (12)	43 (25)		6 (17)	13 (38)	
MRD negative (10^–5^)^‡^	*n* = 193	*n* = 176		*n* = 35	*n* = 35	
* n* (%)	63 (33)	15 (9)	<0.0001	9 (26)	0	0.0022
Sustained MRD negativity (≥6 months), *n* (%)	35 (18)	2 (1)	<0.0001	1 (3)	0	
Sustained MRD negativity (≥12 months), *n* (%)	27 (14)	1 (1)	<0.0001	1 (3)	0	

*MRD* minimal residual disease, *D-Rd* daratumumab plus lenalidomide/dexamethasone, *Rd* lenalidomide/dexamethasone, *ORR* overall response rate, *CR* complete response, *VGPR* very good partial response, *PR* partial response, *ITT* intent-to-treat.

*Patients with high cytogenetic risk had a *t*(4;14), *t*(14;16), or del17p abnormality.

^†^Response-evaluable population.

^‡^ITT population.

At a sensitivity threshold of 10^–5^, MRD-negativity rates were higher with D-Rd compared with Rd, regardless of cytogenetic risk (Table 3). Nine patients with high-risk cytogenetics in the D-Rd group achieved MRD negativity; however, only one of these patients was able to sustain this response. No patients in the Rd group who had a high-risk cytogenetic feature achieved MRD negativity. MRD negativity was sustained for at least 6 months and at least 12 months in 18% and 14% of patients treated with D-Rd, respectively, versus 1% of patients treated with Rd in the standard cytogenetic risk subgroup (Table 3).

**Fig. 2:**
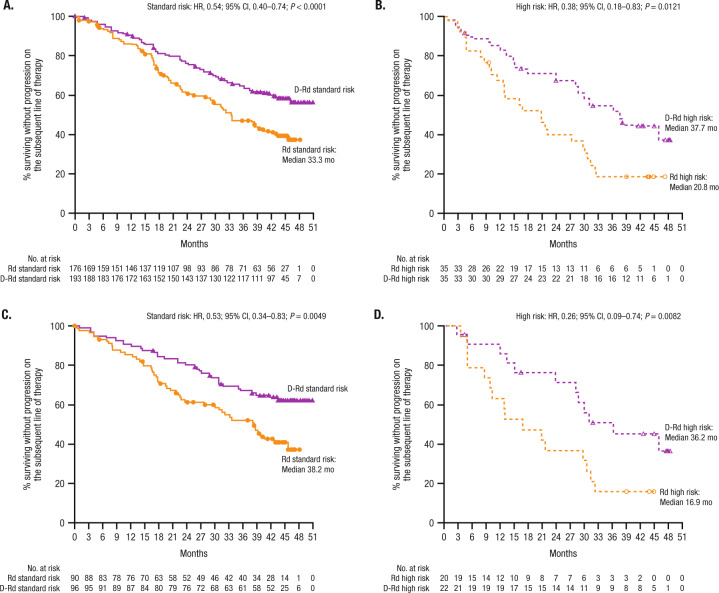
PFS2 based on cytogenetic risk status. PFS2 in the ITT/biomarker risk population:* **A** standard and **B** high cytogenetic risk patients. PFS2 in patients with one prior line of therapy: **C** standard and **D** high cytogenetic risk patients. PFS2 progression-free survival on the next subsequent line of therapy, D-Rd daratumumab plus lenalidomide/dexamethasone, Rd lenalidomide/dexamethasone, HR hazard ratio, CI confidence interval, ITT intent-to-treat. *Patients in the ITT population who met the biomarker criteria for risk assessment.

D-Rd prolonged PFS2 versus Rd in both standard cytogenetic risk (median: NE vs 33.3 months; HR, 0.54; 95% CI, 0.40–0.74; *P* < 0.0001; Fig. 2A) and high cytogenetic risk (median: 37.7 months vs 20.8 months; HR, 0.38; 95% CI, 0.18–0.83; *P* = 0.0121) subgroups (Fig. 2B). Among patients with one prior line of therapy, PFS2 was prolonged with D-Rd versus Rd in patients with standard cytogenetic risk (median: NE vs 38.2 months; HR, 0.53; 95% CI, 0.34–0.83; *P* = 0.0049; Fig. 2C) and high cytogenetic risk (median: 36.2 months vs 16.9 months; HR, 0.26; 95% CI, 0.09–0.74; *P* = 0.0082; Fig. 2D).

At the time of the analysis, among patients with standard cytogenetic risk, 71 (36.8%) deaths were observed in the D-Rd group versus 68 (38.6%) deaths in the Rd group. Among patients with high cytogenetic risk, 18 (51.4%) deaths were observed in the D-Rd group versus 22 (62.9%) deaths in the Rd group. The overall survival data were immature, and follow-up for overall survival is ongoing.

### Safety

The most common (≥25% of patients) any-grade treatment-emergent adverse events (TEAEs) and most common (≥5% of patients) grade 3/4 TEAEs are summarized in Table 4. The most common hematologic adverse event was neutropenia, followed by anemia and thrombocytopenia. The most common infection was upper respiratory tract infection, followed by nasopharyngitis and pneumonia.

**Table 4:** Most common any-grade (≥25% of patients) and Grade 3/4 (≥5% of patients) TEAEs.

	Any grade				Grade 3/4			
	Standard risk		High risk*		Standard risk		High risk*	
TEAE, *n* (%)	D-Rd (*n* = 192)	Rd (*n* = 176)	D-Rd (*n* = 35)	Rd (*n* = 34)	D-Rd (*n* = 192)	Rd (*n* = 176)	D-Rd (*n* = 35)	Rd (*n* = 34)
*Hematologic*								
Neutropenia	116 (60)	80 (46)	22 (63)	18 (53)	103 (54)	69 (39)	17 (49)	16 (47)
Febrile neutropenia	10 (5)	4 (2)	2 (6)	0	10 (5)	4 (2)	2 (6)	0
Anemia	71 (37)	62 (35)	13 (37)	15 (44)	31 (16)	32 (18)	7 (20)	10 (29)
Thrombocytopenia	55 (29)	46 (26)	13 (37)	14 (41)	25 (13)	24 (14)	8 (23)	10 (29)
Leukopenia	18 (9)	15 (9)	3 (9)	4 (12)	6 (3)	4 (2)	1 (3)	3 (9)
Lymphopenia	10 (5)	10 (6)	5 (14)	3 (9)	8 (4)	7 (4)	5 (14)	3 (9)
*Nonhematologic*								
Diarrhea	104 (54)	53 (30)	18 (51)	16 (47)	20 (10)	5 (3)	3 (9)	4 (12)
Upper respiratory tract infection	82 (43)	56 (32)	10 (29)	8 (24)	4 (2)	3 (2)	0	0
Fatigue	74 (39)	52 (30)	12 (34)	12 (35)	13 (7)	9 (5)	4 (11)	2 (6)
Cough	65 (34)	23 (13)	11 (31)	6 (18)	0	0	0	0
Nasopharyngitis	64 (33)	37 (21)	11 (31)	9 (27)	0	0	0	0
Constipation	57 (30)	46 (26)	12 (34)	10 (29)	0	1 (1)	2 (6)	1 (3)
Insomnia	54 (28)	41 (23)	9 (26)	4 (12)	5 (3)	3 (2)	0	1 (3)
Muscle spasms	52 (27)	36 (21)	12 (34)	7 (21)	2 (1)	3 (2)	1 (3)	0
Pneumonia	51 (27)	30 (17)	10 (29)	6 (18)	32 (17)	20 (11)	4 (11)	4 (12)
Nausea	48 (25)	37 (21)	14 (40)	8 (24)	5 (3)	2 (1)	1 (3)	0
Peripheral edema	47 (25)	24 (14)	8 (23)	3 (9)	1 (1)	1 (1)	0	0
Pyrexia	48 (25)	18 (10)	10 (29)	5 (15)	5 (3)	5 (3)	1 (3)	1 (3)
Dyspnea	37 (19)	23 (13)	7 (20)	4 (12)	2 (1)	1 (1)	4 (11)	0
Hypokalemia	37 (19)	19 (11)	7 (20)	4 (12)	14 (7)	5 (3)	2 (6)	1 (3)
Cataract	35 (18)	24 (14)	4 (11)	2 (6)	13 (7)	8 (5)	1 (3)	1 (3)
Bronchitis	32 (17)	24 (14)	10 (29)	4 (12)	6 (3)	5 (3)	0	0
Arthralgia	27 (14)	22 (13)	10 (29)	5 (15)	2 (1)	1 (1)	1 (3)	0
Influenza	20 (10)	9 (5)	5 (14)	2 (6)	2 (1)	0	2 (6)	0
Hyperglycemia	18 (9)	14 (8)	6 (17)	3 (9)	5 (3)	7 (4)	4 (11)	2 (6)
Hypophosphatemia	12 (6)	7 (4)	4 (11)	2 (6)	9 (5)	5 (3)	3 (9)	0
Hypertension	12 (6)	8 (5)	9 (26)	2 (6)	5 (3)	2 (1)	4 (11)	0
Increased alanine aminotransferase	12 (6)	7 (4)	5 (14)	3 (9)	6 (3)	2 (1)	2 (6)	1 (3)
Syncope	9 (5)	2 (1)	2 (6)	1 (3)	9 (5)	2 (1)	2 (6)	1 (3)
Pulmonary embolism	4 (2)	8 (5)	2 (6)	2 (6)	4 (2)	7 (4)	2 (6)	2 (6)
Hypercalcemia	2 (1)	6 (3)	3 (9)	2 (6)	1 (1)	2 (1)	2 (6)	2 (6)

*TEAE* treatment-emergent adverse event, *D-Rd* daratumumab plus lenalidomide/dexamethasone, *Rd* lenalidomide/dexamethasone.

*Patients with high cytogenetic risk had a *t*(4;14), *t*(14;16), or del17p abnormality.

## Discussion

After more than 3 years of follow-up, D-Rd continued to demonstrate improved efficacy versus Rd alone in patients with RRMM regardless of cytogenetic risk status; however, as has been observed previously for high-risk patients, this subgroup had poorer outcomes in both treatment arms. At a median follow-up of 44.3 months, D-Rd reduced the risk of disease progression or death by 57% versus Rd alone in patients with standard cytogenetic risk and by 66% in patients with high cytogenetic risk. Median PFS for patients treated with D-Rd was NE in those with standard cytogenetic risk (vs 18.6 months with Rd; *P* < 0.0001) and was 26.8 months in those with high cytogenetic risk (vs 8.3 months with Rd; *P* = 0.0035). Similar results were observed in the subset of patients with one prior line of therapy. Deep responses were observed with D-Rd in both patients with standard cytogenetic risk (≥CR: 58% vs 25% with Rd) and high cytogenetic risk (≥CR: 43% vs 9% with Rd). Time to reach VGPR or better and CR or better was decreased with D-Rd compared with Rd in both the standard and high cytogenetic risk subgroups. Regardless of cytogenetic risk, MRD-negativity (10^–5^) rates were higher with D-Rd versus Rd, and more patients treated with D-Rd achieved a sustained MRD response, which translates into better patient outcomes^15^, suggesting that achievement of MRD negativity can predict clinical benefit across all patient risk groups. In addition, PFS2 was prolonged with D-Rd compared with Rd in the ITT population and in patients with one prior line of therapy, regardless of cytogenetic risk status. Of note, although favorable outcomes were achieved by D-Rd in patients with high cytogenetic risk, clinical benefits were of lesser magnitude than in patients with standard cytogenetic risk, demonstrating that D-Rd reduces but does not abrogate the adverse impact of high-risk cytogenetics.

This extended follow-up of POLLUX complements the results reported after a median follow-up of 25.4 months^14^. In the earlier analysis, D-Rd reduced the risk of disease progression or death by 70% versus Rd alone in patients with standard cytogenetic risk (D-Rd, *n* = 133; Rd, *n* = 113) and by 47% in patients with high cytogenetic risk (D-Rd, *n* = 28; Rd, *n* = 37), with cytogenetic abnormalities assessed by central next-generation sequencing^14^. The next-generation sequencing and FISH methods have been shown to have high concordance (88%–98%) in identifying high-risk cytogenetics^16^.

The safety profile of D-Rd in the standard and high cytogenetic risk subgroups was consistent with the overall population of POLLUX^9,10^. No new safety concerns were observed following a median follow-up of more than 3 years.

Although cross-trial comparisons are limited by differences in risk-group definitions (due to lack of consensus criteria on cutoffs), patient populations, and clinical settings, they allow for a comprehensive assessment of a treatment regimen’s clinical benefit. The efficacy results in terms of HR of PFS from this study are similar to those for other studies of the immunomodulatory drug (IMiD)–containing regimens in patients with RRMM and high cytogenetic risk. In a subgroup analysis of the phase 3 ASPIRE study, among patients with high cytogenetic risk (defined as the presence of *t*[4;14], *t*[14;16], or del17p according to FISH), median PFS was 23.1 months with carfilzomib plus Rd versus 13.9 months with Rd alone (HR, 0.70; 95% CI, 0.43–1.16; *P* = 0.0829)^17^. In the phase 3 study of elotuzumab (ELOQUENT-2), at a median follow-up of 46 months, median PFS among high-risk patients (defined as ISS stage II or III disease and a t[4;14] or del17p abnormality) was 15 months with elotuzumab plus Rd compared with 7 months with Rd alone (HR, 0.64; 95% CI, 0.43–0.97; *P* = 0.0331)^18^. In a post hoc analysis of the TOURMALINE-MM1 study according to cytogenetic risk status, median PFS in high-risk patients (defined as *t*[4;14], *t*[14;16] or del17p abnormality) was 21.4 months with ixazomib plus Rd versus 9.7 months with Rd alone (HR, 0.543; 95% CI, 0.321–0.918; *P* = 0.021)^19^. None of these analyses reported the effects of these combinations on MRD negativity according to cytogenetic risk status.

Improvement in PFS with a daratumumab-based regimen versus standard of care was also observed in the phase 3 CASTOR study of daratumumab plus Vd (D-Vd) versus Vd alone in RRMM, regardless of cytogenetic risk status^20^. In an updated subgroup analysis after a median follow-up of more than 3 years, treatment with D-Vd prolonged PFS compared with Vd in patients with standard cytogenetic risk (16.6 vs 6.6 months; HR, 0.26; 95% CI, 0.19–0.37) as well as those with high cytogenetic risk (12.6 vs 6.2 months; HR, 0.41; 95% CI, 0.21–0.83)^21^. The benefits of D-Vd over Vd in the high-risk population were not only maintained but strengthened, demonstrating improved clinical benefit in terms of PFS, ORR, rate of VGPR or better, and MRD-negativity rate. Moreover, among high-risk patients, MRD negativity was only achieved in patients treated with D-Vd.

Although high cytogenetic risk patients in the POLLUX study were defined using the 2009 IMWG criteria available at the time of study design instead of the updated 2016 criteria, the definition used to determine high cytogenetic risk status (*t*[4;14], *t*[14;16], or del17p abnormality) is consistent with the previously mentioned studies. Additionally, the fact that among the patients with high cytogenetic risk in this study, patients who received Rd had a lower PFS further confirms the correct categorization of patients.

This subgroup analysis was limited by incomplete cytogenetic abnormality data collected for patients enrolled in the POLLUX study; cytogenetic testing was not performed in 23% of patients. Also, the cytogenetic testing for *t*(4;14), *t*(14;16), and del17p abnormalities was performed locally, and threshold levels for a positive finding were not uniform for all patients. Although additional abnormalities were requested, they were not required by the protocol and were not always reported. About two-thirds of patients with high cytogenetic risk in this study had del17p abnormalities. The presence of *TP53* mutation status in del17p patients is known to adversely affect clinical outcomes; however, details on *TP53* mutational status were not collected and thus a potential for bias based on *TP53* mutation status cannot be ruled out. Small sample sizes in the cytogenetic risk subgroups precluded us from conducting additional subgroup analyses. MRD results were not available in 22% of patients with CR or better. Patients without MRD assessment were considered as MRD positive, potentially underestimating the rate of MRD negativity.

In conclusion, the results from this updated subgroup analysis of POLLUX show that D-Rd in comparison with the standard of care alone improves the outcome of patients with high-risk RRMM. Moreover, the analysis of all the previously mentioned trials confirms that daratumumab is effective in patients with MM, regardless of cytogenetic risk status.
